# Improved Efficiency of All-Inorganic Quantum-Dot Light-Emitting Diodes via Interface Engineering

**DOI:** 10.3389/fchem.2020.00265

**Published:** 2020-04-23

**Authors:** Qiulei Xu, Xinyu Li, Qingli Lin, Huaibin Shen, Hongzhe Wang, Zuliang Du

**Affiliations:** Key Lab for Special Functional Materials, Ministry of Education, National and Local Joint Engineering Research Center for High-Efficiency Display and Lighting Technology, Collaborative Innovation Center of Nano Functional Materials and Applications, School of Materials Science and Engineering, Henan University, Kaifeng, China

**Keywords:** NiO_**x**_, all-inorganic, quantum dots, light-emitting devices, high efficiency

## Abstract

As the charge transport layer of quantum dot (QD) light-emitting diodes (QLEDs), metal oxides are expected to be more stable compared with organic materials. However, the efficiency of metal oxide-based all-inorganic QLEDs is still far behind that of organic–inorganic hybrid ones. The main reason is the strong interaction between metal oxide and QDs leading to the emission quenching of QDs. Here, we demonstrated nickel oxide (NiO_x_)-based all-inorganic QLEDs with a maximum current efficiency of 20.4 cd A^−1^ and external quantum efficiency (EQE) of 5.5%, which is among the most efficient all-inorganic QLEDs. The high efficiency is mainly attributed to the aluminum oxide (Al_2_O_3_) deposited at the NiO_x_/QDs interface to suppress the strong quenching effect of NiO_x_ on the QD emission, together with the molybdenum oxide (MoO_x_) that reduced the leakage current and facilitated hole injection, more than 300% enhancement was achieved compared with the pristine NiO_x_-based QLEDs. Our study confirmed the effect of decorating the NiO_x_/QDs interface on the performance enhancement of the all-inorganic QLEDs.

## Introduction

Quantum dots (QDs) have many advantages including high color purity, high photoluminescence (PL) quantum yield (QY), and high stability, which make them promising luminescent materials for light-emitting diodes (LEDs) (Anikeeva et al., 2009; Bae et al., 2013; Shirasaki et al., 2013; Shen et al., 2015; Chen et al., 2018; Cao et al., 2019; Zhang et al., 2019). Recently, the performance of QD LEDs (QLEDs) has been improved greatly, the external quantum efficiencies (EQEs) for tricolor QLEDs have all surpassed 20%, with peak EQEs of 30.4% for red, 22.9% for green, and 19.8% for blue QLEDs, respectively (Wang et al., 2017; Shen et al., 2019; Song et al., 2019). At present, highly efficient QLEDs are mainly based on hybrid organic–inorganic structure, in which poly(3,4-ethylenedioxythiophene) polystyrene sulfonate (PEDOT:PSS) is widely used as the hole injection layer (HIL); poly[N,N'-bis(4-butylphenyl)-N,N'-bis(phenyl)benzidine] (Poly-TPD), poly{9,9-dioctylfluorene-co-N-[4-(3-methylpropyl)]diphenylamine} (TFB), or poly(N-vinyl carbazole) (PVK) are adopted as the hole transport layer (HTL); and zinc oxide (ZnO) nanoparticles (NPs) are used as the electron transport layer (ETL) (Qian et al., 2011; Dai et al., 2014; Zhang et al., 2019). As we know, organic materials are sensitive to moisture and may degrade under high operating currents, which affect the stability of devices, and consequently, the strict encapsulation technology is indispensable. To solve this problem, it is necessary to seek more stable hole transport materials that can endure a high carrier density at high luminance. Many inorganic metal oxides [nickel oxide (NiO_x_), tungsten oxide (WO_x_), molybdenum oxide (MoO_x_), vanadium oxide (VO_x_), etc.] have been applied as HIL in optical electronic devices to improve the device stability (Murase and Yang, 2012; Huu Tuan et al., 2014; Yang et al., 2014; Zhang et al., 2017), and NiO_x_ is a promising hole transport material among them due to its nature of intrinsic p-type semiconductor with a wide bandgap and high transparency. Moreover, NiO_x_ possesses relatively proper band energy for efficient hole injection and electron blocking to confine the excitons in the QD emitting layer [~5.2 eV for the valance band maximum (VBM) and ~1.6 eV for the conductive band minimum (CBM)]. Nevertheless, NiO_x_-based all-inorganic QLEDs with ZnO as ETL exhibited poor efficiency (Mashford et al., 2010), which is mainly attributed to two reasons. First, the higher electron mobility [~10^−2^ cm^2^ (V·s)^−1^] of ZnO NPs and small energy barrier of the conductive band at the QDs/ZnO interface lead to imbalanced carrier transport due to easier electron injection. Second, the excitons formed near the NiO_x_ layer are subject to the surface of NiO_x_, and a large number of free carriers and defects/traps on the surface of adjacent NiO_x_ HTL leads to the quenching of QD emission (Caruge et al., 2006, 2008; Wu and Yeow, 2010). It is reported that many dipolar surface species of NiOOH are present on the solution-processed NiO_x_ films and induce a strong localized electric field, which facilitates radiationless decay channels with a charge-transfer/charging and/or energy transfer processes and leads to a severe decrease of device efficiency (Ratcliff et al., 2011; Liu et al., 2015).

To address this issue, a modification of the NiO_x_/QD interface is needed. Several kinds of buffer layer have been inserted to suppress the exciton quenching induced by NiO_x_. By introducing ultrathin aluminum oxide (Al_2_O_3_) layer at the NiO_x_/QD layer interface (Zhang et al., 2016; Ji et al., 2017, 2018), Ji et al. fabricated highly efficient green all-inorganic QLEDs, in which over 800% enhancement for the current efficiency/EQE of up to 34.1 cd·A^−1^/8.1% was achieved when the Al_2_O_3_ layer was obtained by atomic layer deposition (ALD), and this represented the highest EQE for all-inorganic QLEDs reported ever. With ultrathin lithium fluoride (LiF) being inserted at the NiO_x_/QD interface and ultrathin Al_2_O_3_ being inserted between the QDs and ZnO layer (Yang et al., 2018), Yang et al. reported highly efficient all-inorganic QLEDs with a maximum EQE of 6.52% and a long device life time of 16,120 h at 100 cd·m^−2^. Li et al. reported all-inorganic QLEDs of the highest maximum brightness of 40,000 cd·m^−2^ by sputtering ultrathin MgO at the NiMgO/QD interface; however, the maximum EQE is only 1.5% (Jiang et al., 2019). These results indicate the importance of decoration of the NiO_x_/QD interface on suppressing the QD emission quenching and improving the performance of the all-inorganic QLED efficiency. Among them, the Al_2_O_3_ buffer layer obtained by ALD technology has more advantages since the film thickness can be precisely controlled at atomic level by alternating the exposure cycle of trimethylaluminum [Al(CH_3_)_3_] and H_2_O, and the as-prepared films possess good uniformity over large substrates and excellent conformality on three-dimensional surface topologies. Furthermore, the hydroxyl (-OH) in the NiOOH species can be consumed during the exposure to Al(CH_3_)_3_ deposition cycles. Nevertheless, the maximum EQE for all-inorganic QLEDs with Al_2_O_3_ buffer layer is still very low, which is likely due to imbalanced carrier transport in devices resulting from the inefficient hole injection from indium tin oxide (ITO) to the NiO_x_ layer and the relatively higher energy barrier between the NiO_x_ layer and QD layer. To solve this problem, more researches are still needed to optimize the structure of NiO_x_-based all-inorganic QLEDs and improve the device efficiency.

Here, we demonstrated highly efficient all-inorganic QLEDs with an optimized structure of ITO/solution-processed MoO_x_ (sMoO_x_)/NiO_x_/Al_2_O_3_/QDs/ZnO/Al through all solution-process method except for Al_2_O_3_ layer and the electrodes. The ultrathin Al_2_O_3_ inserted at the NiO_x_/QDs interface was to suppress the strong quenching effect of NiO_x_ on the emission of QD. And the sMoO_x_ introduced before the NiO_x_ layer was aimed to reduce leakage current and facilitate the hole injection from anode to the emitting layer and minimize the hole-blocking effect of Al_2_O_3_ layer. Our resultant all-inorganic QLEDs reached a high current efficiency of 20.4 cd A^−1^ and a maximum EQE of 5.5%, more than 300% enhancement was achieved compared with the pristine NiO_x_-based QLEDs.

## Materials and Methods

### Preparation of Green Quantum Dots and Metal Oxide Solution

Cadmium selenide (CdSe)/zinc sulfide (ZnS) QDs were synthesized according to the method reported in the literature (Li et al., 2019). The QDs in octane solution exhibited a green emission with the PL peak at 525 nm (Supplementary Figure 1). The NiO_x_ precursor was prepared by a modified method (Mashford et al., 2010); the mixture of nickel acetate tetrahydrate [Ni(OAc)_2_·4H_2_O; purchased from Aldrich] and equimolar quantity of monoethanolamine (MEA; purchased from Aldrich) in ethanol was heated at 60°C for 2 h and stirred overnight. 0.1 M MoO_x_ solutions were synthesized by a thermal decomposition method using ammonium heptamolybdate [(NH_4_)_6_Mo_7_O_24_·4H_2_O] as a precursor (Murase and Yang, 2012; Vu et al., 2016). The ZnO NPs were prepared by slowly mixing 0.1 M zinc acetate in dimethyl sulfoxide (DMSO) and 0.3 M tetramethylammonium hydroxide (TMAH) in ethanol together for 1 h, and the ZnO particles were precipitated by adding hexane/ethanol to the solution.

### Fabrication of Quantum Dot Light-Emitting Diode Devices

The all-inorganic QLED structure consists of ITO/MoO_x_/NiO_x_/Al_2_O_3_ (*x* cycles)/QDs/ZnO/Al. The NiO_x_, QDs, and ZnO are used as HTL, emission layer, and ETL, respectively. Before fabricating the devices, the ITO substrates were ultrasonically cleaned in detergent, DI water, acetone, and isopropyl alcohol for 15 min successively followed by an *ex situ* UV ozone treatment in air for 15 min. This as-prepared MoO_x_ precursor solution was spin-coated onto the UV ozone-treated ITO substrates at 4,000 rpm and then baked at 120°C for 10 min to get the MoO_x_ film. Then, the NiO_x_ precursor was spin-coated at 2,000 rpm and annealed at 275°C for 30 min in air to obtain a highly conductive layer. The Al_2_O_3_ layer was deposited by alternating exposures of Al(CH_3_)_3_ and H_2_O with the same substrate and maintaining the temperature at 200°C, and the thickness is approximately 0.1 nm for each ALD cycle. Al_2_O_3_ layers with different thicknesses were deposited on the NiO_x_ films for device A (zero cycle), B (one cycle), C (two cycles), and D (three cycles), respectively. Note that no MoO_x_ layers were inserted for devices A to C, and device A is a control device without the Al_2_O_3_ interlayer. The QD octane solution (18 mg·ml^−1^) was then spin-coated on the NiO_x_/Al_2_O_3_ layer at 2,000 rpm in N_2_-filled glove box. After that, ZnO ethanol solution (30 mg·ml^−1^) was spin-coated at 2,000 rpm and annealed at 60°C for 30 min to remove the residual solvent. Finally, the Al cathode was thermal evaporated in the vacuum chamber at pressure below 4 × 10^−6^ Torr. The Al cathode lines with a width of 2.0 mm were deposited orthogonally to the 2 mm ITO anode lines to form a 4 mm^2^ active area.

### Measurements and Characterization

Current density–voltage–luminance (J–V–L) characteristics of QLEDs were tested using a Keithley 2400 source meter and a picoammeter (Keithley 6485) with a calibrated Newport silicon diode under ambient conditions. The luminance was calibrated using a Minolta luminance meter (CS-100). The electroluminescence spectra were obtained with an Ocean Optics spectrometer (USB2000, relative irradiance mode) and a Keithley 2400 source meter. The room temperature PL spectrum of the QDs in octane was collected by the Ocean Optics Maya 2000-Pro spectrometer under an excitation wavelength of 365 nm. Time-resolved PL (TRPL) measurements were carried out with Edinburgh Instruments FL920 spectrometer, utilizing a 400-nm excitation light source. X-ray photoelectron spectroscopy (XPS) was obtained using a Kratos Axis-Ultra spectrometer with a monochromatic Al Kα source, 15 kV/8 mA. The atomic force microscopy (AFM) images were recorded in the tapping mode by Bruker Multimode-8. The UV photoelectron spectroscopy (UPS; Thermo Scientific ESCALAB 250 XI) measurement was performed using a He I discharge lamp (hv = 21.22 eV) under high vacuum (2.5 × 10^−8^ mbar) and the UPS spectra of MoO_x_ and NiO_x_ was meaured (Supplementary Figure 4).

## Results and Discussion

**Figure 1:**
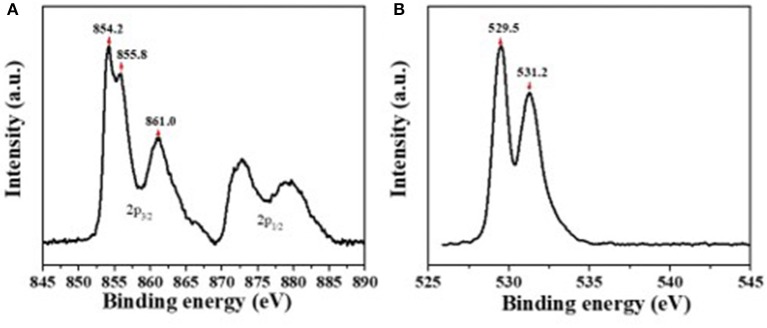
The X-ray photoelectron spectroscopy (XPS) spectra for Ni 2p **(A)** and O1s **(B)**.

The composition of the solution-processed NiO_x_ films was studied by XPS analysis. Figure 1A shows the XPS spectrum for Ni 2p_3/2_ state possessing three peaks. The first peak centered at a binding energy of 854.2 eV corresponds to Ni^2+^ in the standard Ni-O octahedral bonding configuration in cubic rock salt NiO_x_. The adjacent peak shoulder located at 855.9 eV was ascribed to Ni^2+^ vacancy-induced Ni^3+^ ion and NiOOH (Sasi and Gopchandran, 2007; Manders et al., 2013). The broad peak centered at 861.0 eV has been ascribed to a shake-up process in the NiO structure. Figure 1B shows the XPS spectrum for the O 1s state, the peak centered at 529.5 eV confirms the Ni-O octahedral bonding in NiO_x_. The peak at 531.2 eV is indicative of nickel hydroxides and oxyhydroxides, including defective NiO_x_ with hydroxyl groups adsorbed on the surface (Han et al., 2006; Ratcliff et al., 2011).

**Figure 2:**
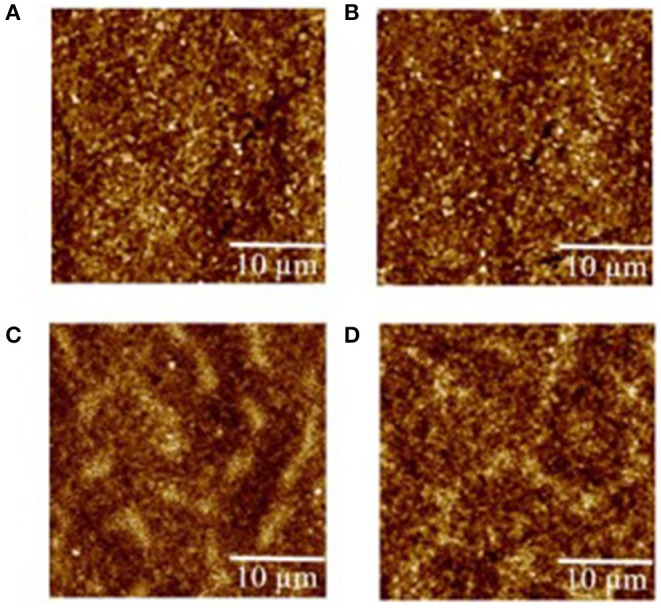
Atomic force microscopy (AFM) images of **(A)** indium tin oxide (ITO)/nickel oxide (NiO_x_), **(B)** ITO/NiO_x_/aluminum oxide (Al_2_O_3_) (two cycles), **(C)** ITO/NiO_x_/Al_2_O_3_ (two cycles)/quantum dots (QDs), and **(D)** ITO/NiO_x_/Al_2_O_3_ (two cycles)/QDs/zinc oxide (ZnO). The corresponding root-mean-square (RMS) values of the samples are 1.23, 1.24, 1.51, 2.12 nm, respectively.

The morphology evolution of each layer within the QLEDs was assessed by atomic force microscopy (AFM; Figure 2). The root-mean-square (RMS) roughness of pure ITO (Supplementary Figure 2) is 2.38 nm, and the value decreased to 1.23 nm after the spin-coating of NiO_x_, which suggested that the ITO substrate was smoothed. The following ultrathin Al_2_O_3_ deposition (second cycle) had little effect on the roughness of the NiO_x_ film. Then, the RMS roughness for substrate increased slightly as the layer number increased, which showed 1.51 nm for QD layer and 2.12 nm for ZnO layer, respectively.

**Figure 3:**
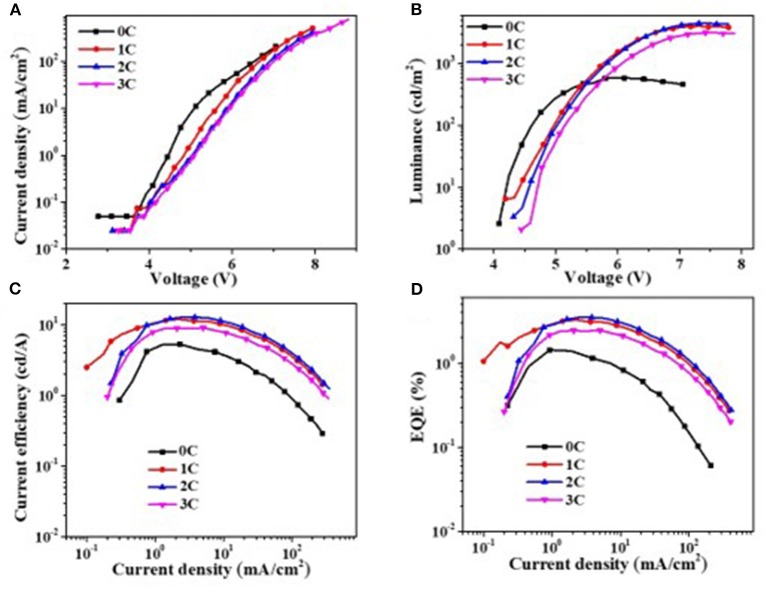
Photoelectric properties of quantum dot light-emitting diodes (QLEDs). **(A)** Voltage vs. current density (V–J), **(B)** voltage vs. luminance (V–L), **(C)** current density–luminous efficiency, and **(D)** current density–external quantum efficiency. 0C means 0 cycle deposition of aluminum oxide (Al_2_O_3_), each cycle is about 0.1 nm.

Since Al_2_O_3_ is an insulating material, it is very important to control its thickness precisely *via* the ALD process. To get the optical thickness of Al_2_O_3_, we first fabricated all-inorganic QLEDs consisting of a structure of ITO/NiO_x_/Al_2_O_3_ (n cycle)/QDs/ZnO/Al. Different deposition cycles of Al_2_O_3_ (0C, 1C, 2C, 3C) were applied at the NiO_x_/QD interface, and the corresponding photoelectrical properties of devices were characterized and shown in Figure 3. Al_2_O_3_ showed a remarkable influence on the performance of all-inorganic QLEDs. The current density decreased evidently with the increasing thickness of Al_2_O_3_ at a given voltage. For example, the current density for devices with even one cycle (0.1 nm) of Al_2_O_3_ deposition dropped to 1.4 mA·cm^−2^ at 5 V, which is four times lower than that of the QLEDs without the Al_2_O_3_ layer. The reduced current density is probably due to the insulated Al_2_O_3_ layer, which limits the hole injection from NiO_x_, and this can be confirmed by the lower hole density in hole-only devices consisting of ITO/NiO_x_/Al_2_O_3_/QDs/MoO_3_/Al than that without the ultrathin Al_2_O_3_ layer (Supplementary Figure 3). The turn-on voltage slightly increased from 4.1 V (0 C) to 4.4 V (3 C) with the increasing thickness of Al_2_O_3_ layer. Despite the lower current density and higher turn-on voltage, the QLEDs with Al_2_O_3_ passivated layer exhibited more than 600% enhancement in luminance and more than 200% improvement in current efficiency and EQE (see Supplementary Table 1 in supporting information), which suggested that the emission quenching induced by NiO_x_ played a more critical role in deterring the performance of NiO_x_-based all-inorganic QLEDs. Particularly, devices with two cycles of Al_2_O_3_ deposition showed the highest current efficiency/maximum EQE of 12.8 cd A^−1^/3.5% at 5.5 V, respectively.

**Figure 4:**
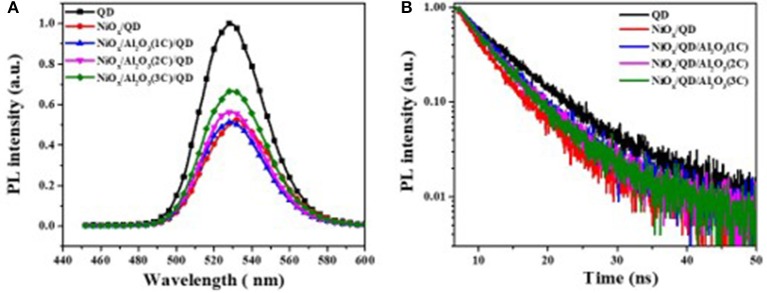
**(A)** Steady-state and **(B)** time-resolved photoluminescence (PL) spectra of samples with and without the aluminum oxide (Al_2_O_3_) layer.

To study the effect of ultrathin Al_2_O_3_ layer on the improvement of QLEDs performance, five samples were prepared, namely, F1: Glass/QDs, F2: ITO/NiO_x_/QDs, F3: ITO/NiO_x_/Al_2_O_3_ (1C)/QDs, F4: ITO/NiO_x_/Al_2_O_3_ (2C)/QDs/ZnO, and F5: ITO/NiO_x_/Al_2_O_3_ (3C)/QDs/ZnO, to measure their steady-state and time-resolved PL spectroscopy (as shown in Figure 4). The exciton lifetimes for different film samples were summarized in Table 1. It can be seen that the emission of QD film on glass substrate was peaked at 528 nm with an exciton lifetime of 7.3 ns, while that on NiO_x_ substrate red-shifted to 532 nm and the corresponding emission intensity and exciton lifetime decreased remarkably due to the interaction between QDs and NiO_x_. For samples from F3 to F5, the PL peak blue-shifted to the original location of QD film (528 nm) with Al_2_O_3_ insertion, and the emission intensity and lifetime also showed an obvious increase, which confirmed the positive effect on passivating the surface of the NiO_x_ layer and suppressing the emission quenching induced by NiO_x_ through the introduction of the Al_2_O_3_ layer, and such results were consistent with the previously reported findings.

**Table 1:** Summary of the PL peak and the decay lifetime for different samples.

**Sample**	**PL Peak (nm)**	**Lifetime (ns)**
QD in octane	525	
F1: Glass/QDs	528	7.3
F2: ITO/NiO_x_/QDs	532	5.2
F3: ITO/NiO_x_/Al_2_O_3_ (1C)/QDs	528	5.8
F4: ITO/NiO_x_/Al_2_O_3_ (2C)/QDs	528	6.0
F5: ITO/NiO_x_/Al_2_O_3_ (3C)/QDs	528	6.0

*Al_2_O_3_, aluminum oxide; ITO, indium tin oxide; NiO_x_, nickel oxide; PL, photoluminescence; QD, quantum dot*.

**Figure 5:**
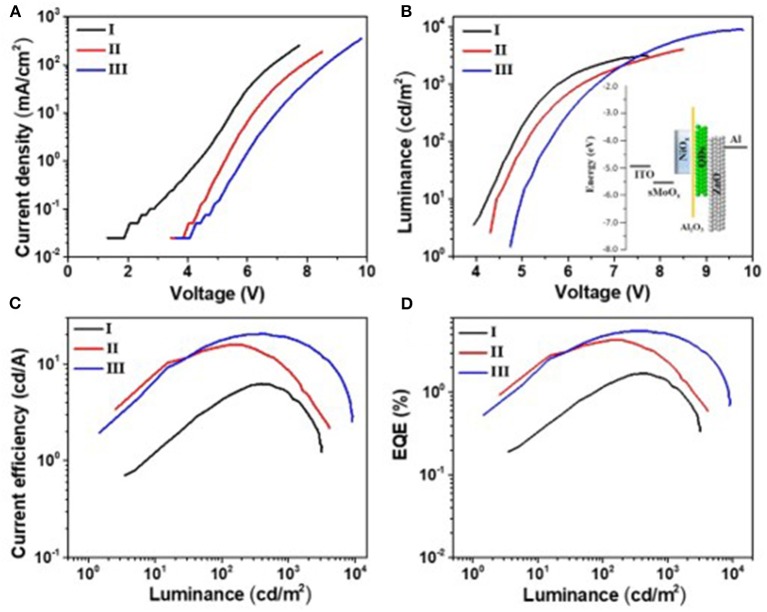
Photoelectric properties of devices I, II, and III. **(A)** Voltage vs. current density (*V–J*), **(B)** voltage vs. luminance (*V–L*), **(C)** luminance–current efficiency, and **(D)** luminance–external quantum efficiency. The inset in **(B)** is the energy level diagrams of quantum dot light-emitting diode (QLED) III.

**Figure 6:**
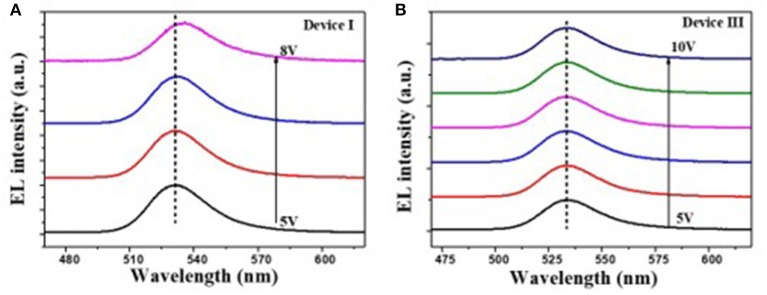
Electroluminescence (EL) spectra of devices I **(A)** and III **(B)** under different voltages.

It is reported that the sMoO_x_ film showed a higher work function of 5.6 eV, better transparency, and smoother surface morphology, providing the QLEDs with good Ohmic contact and small charge transfer resistance (He et al., 2013; Vu et al., 2016). The device structure was further optimized by using sMoO_x_ as HIL to expect an even better device performance. Three kinds of QLEDs were fabricated with structures of ITO/NiO_x_/QDs/ZnO ITO/Al, ITO/sMoO_x_/NiO_x_/QDs/ZnO, and ITO/sMoO_x_/NiO_x_/Al_2_O_3_ (two cycles)/QDs/ZnO/Al for device I, device II, and device III, respectively. The related optoelectronic characteristic curves were shown in Figure 5, and the corresponding performance parameters were summarized in Table 2. Remarkably, device II with sMoO_x_ layer showed a maximum current efficiency of 15.9 cd A^−1^ and an EQE of 4.3%, which was more than two times higher of that of device I (7.5 cd A^−^1/1.7%), and this suggested that the sMoO_x_ was comparable to the ultrathin Al_2_O_3_ in improving the efficiency of NiO_x_-based QLEDs. The device performance improvement for device II can be ascribed to the sMoO_x_ modified layer, which reduced the leakage current and led to a more balanced carrier injection in emitting layer. For device III possessing sMoO_x_ layer as well as two cycles of Al_2_O_3_ layer, the maximum luminance was further improved to 9,140 cd m^−2^ at 9.7 V, which was about 1.8 times of that of device II (4,930 cd m^−2^ at 8.5 V). The maximum current efficiency and EQE for device III were 20.4 cd A^−1^ and 5.5%, respectively, which were about 1.2 times of that of device II. The relatively higher increase in luminance reconfirmed the importance of the Al_2_O_3_ layer in maintaining the high emitting efficiency of the QD layer. The maximum efficiency for device III was obtained at higher-voltage regime, which meant that charge transport became more balanced at higher driving voltage and better tolerance to higher operating voltage for device III than the other two. It is also confirmed from the EL spectra under increasing driving voltage of devices I and III (Figure 6). The EL peak for device I without Al_2_O_3_ layer exhibited a red shift of 4 nm as the voltage increased to 8 V, while that for device III kept its profile from 5 to 10 V. Despite the slightly higher turn-on voltage, the insertion of sMoO_x_ layer combining Al_2_O_3_ layer in NiO_x_-based all-organic QLEDs improved not only the device efficiency but also the performance stability. A comparison of the performance of all-inorganic QLEDs between our work and others in literature was summarized (see Supplementary Table 2).

**Table 2:** Summary of the electrical properties of the QLEDs.

**Device**	****λ_max_** (nm)**	**V_**T**_ (V)**	**L_**max**_ (cd/m^**2**^)**	**EQE_**max**_ (%)**	**η_**Amax**_ (cd/A)**	**η_**Pmax**_ (lm/W)**
I	532	3.9	3,786 (7.6 V)	1.7 (5.3 V)	7.5 (5.3 V)	4.5 (5.3 V)
II	534	4.3	4,930 (8.5 V)	4.3 (5.2 V)	15.9 (5.2 V)	9.6 (5.2 V)
III	534	4.7	9,140 (9.0 V)	5.5 (6.2 V)	20.4 (6.2 V)	10.7 (5.8 V)

*EQE, external quantum efficiency; QLED, quantum dot light-emitting diode*.

## Conclusion

All-inorganic QLEDs with high efficiency were fabricated using solution-processed NiO_x_ as the HTL and ZnO as the ETL, and ultrathin Al_2_O_3_ was deposited at the NiO_x_/QDs interface by the ALD process to reduce the strong quenching effect of NiO_x_ on the QD emission. The corresponding all-inorganic QLEDs exhibited a maximum current efficiency of 19.8 cd A^−1^ and EQE of 4.5%, which is 260% enhancement compared with the QLEDs without Al_2_O_3_ insertion, making them among the highest efficient inorganic QLEDs. This result suggests that the Al_2_O_3_ passivating layer is critical to device efficiency improvement by suppressing QDs emission quenching induced by NiO_x_. Despite great device improvement, the maximum EQE for NiO_x_ all-inorganic QLEDs is still below 10%, which is probably due to the relatively lower hole mobility of NiO_x_ and higher energy barrier for hole transfer from NiO_x_ to the QD layer, resulting in an imbalanced charge injection in devices. The energy level regulating as well as improving electrical performance of NiO_x_ are vital strategies to fabricate high-performance all-inorganic QLEDs.

## Data Availability Statement

All datasets generated for this study are included in the article/Supplementary Material.

## Author Contributions

All authors listed have made a substantial, direct and intellectual contribution to the work, and approved it for publication.

## Conflict of Interest

The authors declare that the research was conducted in the absence of any commercial or financial relationships that could be construed as a potential conflict of interest.
