# Supplementary material for: Improved Efficiency of All-Inorganic Quantum-Dot Light-Emitting Diodes via Interface Engineering
Source: Front Chem. 2020 Apr 23;8:265. doi: 10.3389/fchem.2020.00265 (PMC7191064; doi:10.3389/fchem.2020.00265)
Supplement: Supplementary file 1 [file Table_1.DOCX]

Supplementary Material


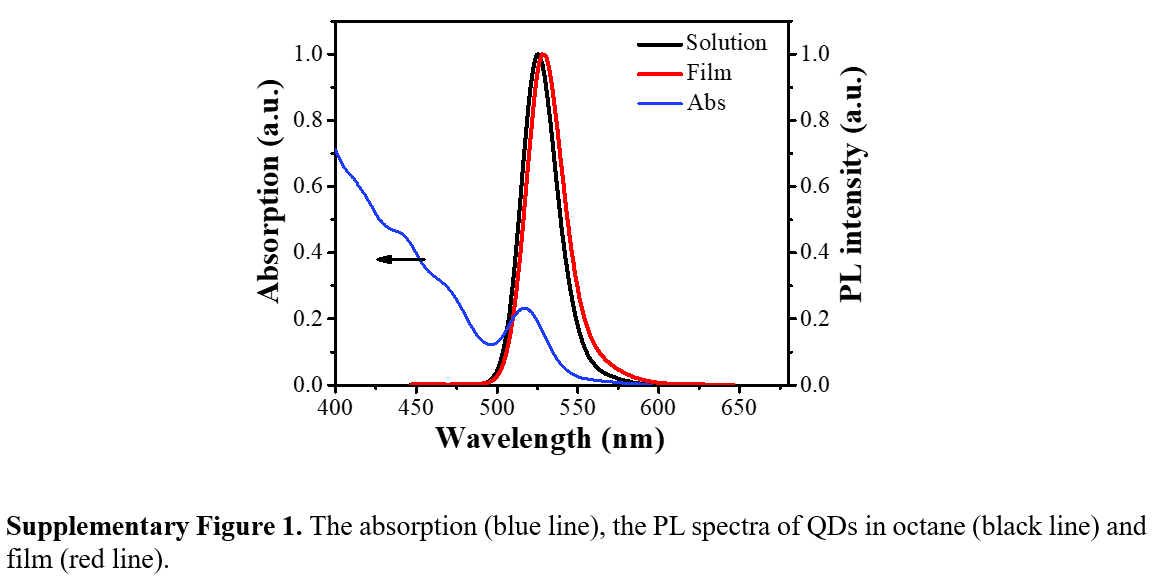


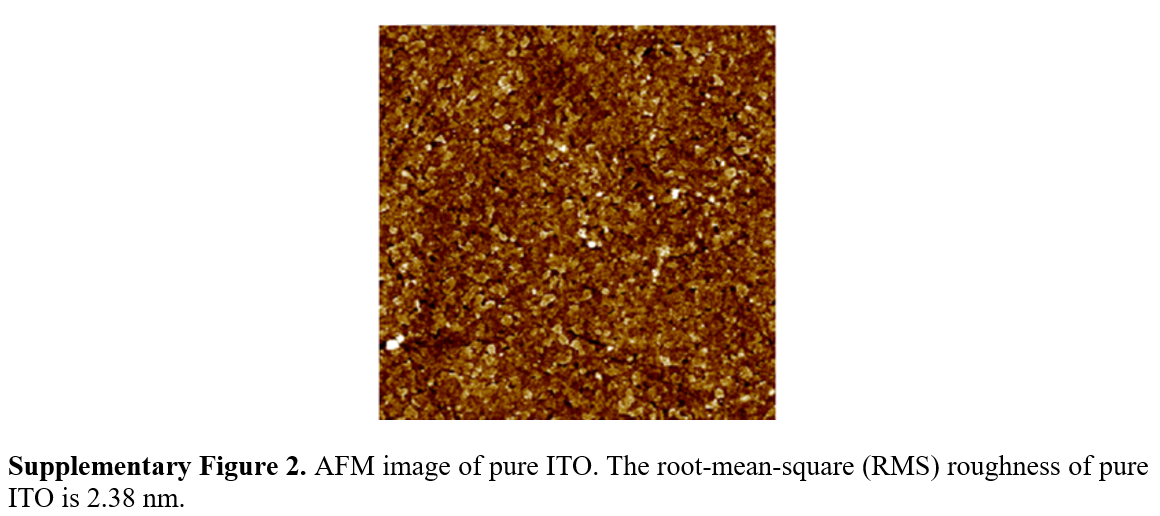


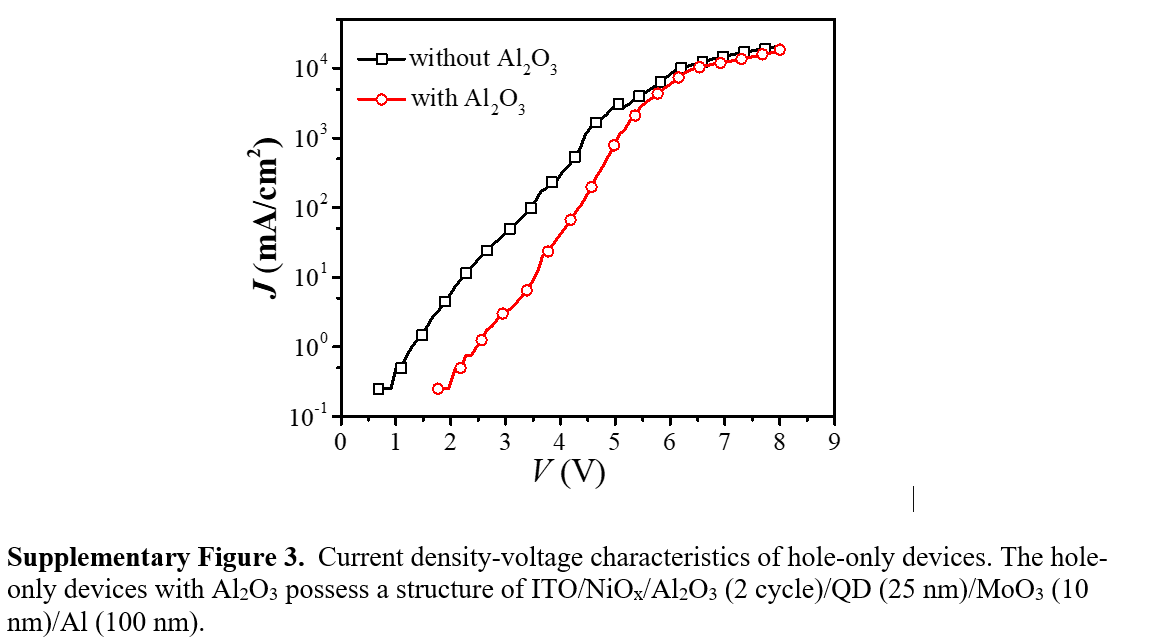


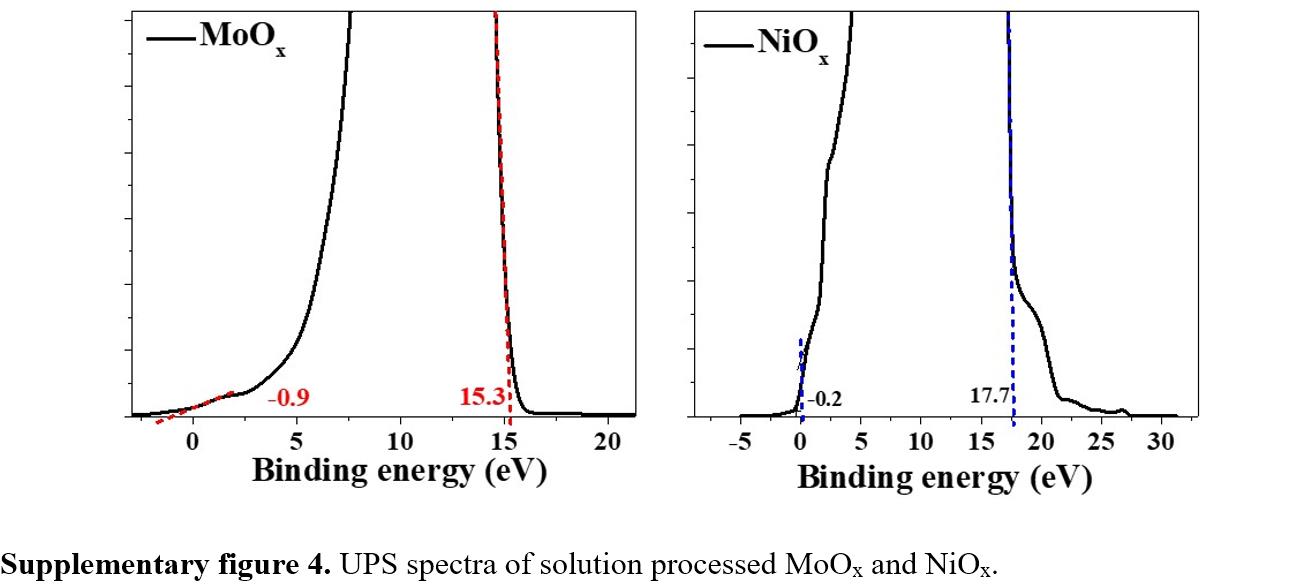


**Supplementary Table 1**. Summary of the electrical properties of the QLEDs with different cycles of Al_2_O_3_.

| device | V_T_ (V) | L_max_ (cd/m^2^) | EQE_max_ (%) | *η*_A_ (cd/A) | *η*_P_ (lm/W) |
| --- | --- | --- | --- | --- | --- |
| 0C | 4.1 | 590 (5.9 V) | 1.4 (4.4 V) | 5.4 (4.4 V) | 3.7 (4.4 V) |
| 1C | 4.2 | 4070 (7.3 V) | 3.3 (5.2 V) | 12.0 (5.2 V) | 7.2 (5.2 V) |
| 2C | 4.3 | 4540 (7.5 V) | 3.5 (5.5 V) | 12.8 (5.5 V) | 7.5 (5.4 V) |
| 3C | 4.4 | 3120 (7.9 V) | 2.4 (5.3 V) | 9.0 (5.7 V) | 5.3 (5.2 V) |

**Supplementary Table 2.** Comparison of the performance of all-inorganic QLEDs.

| **Device Structure** | **Peak Emission**  **[nm]** | **EQE**  **[%]** | **Brightness**  **[cd/m^2^]** | **Reference** |
| --- | --- | --- | --- | --- |
| ITO/NiO^1^/QDs/ZnO:SnO_2_/Al | 638 | 0.1 | 1950 | Nano Letters 2006, 6 (12), 2991–2994 |
| ITO/NiO^2^/QDs/ZnO/Al | 628 | 0.01 | 249 | J. Mater. Chem. 2009, 20 (1), 167–172 |
| ITO/NiO^2^/Al_2_O_3_^2^/QDs/ZnO/Al | 530 | **-----** | >2000 | ACS Photonics 2017, 4 (5), 1271–1278. |
| ITO/MoO_3_/NiO^2^/LiF^3^/QDs/Al_2_O_3_^2^/ZnO/Al | 508 | 6.52 | 21600 | Nano Energy 2018, 46, 229–233 |
| ITO/NiO^2^/QDs/ZnO/Al | 533 | **-----** | 4205 | ACS Applied Materials & Interfaces 2018,  10 (17), 14894–14900 |
| ITO/NiO^2^/Al_2_O_3_^4^/QDs/ZnO/Al | 527 | 8.1 | 14713 | Nanoscale 2018, 10 (23), 11103–11109. |
| ITO/Ni_0.88_Mg_0.12_O^1^/MgO^1^/QDs/ZnMgO/Al | 535 | 1.47 | 40000 | ACS Appl. Mater. Interfaces 2019,  11 (12) 11119-11124 |
| ITO/sMoO_3_/NiO_x_^2^/Al_2_O_3_^4^/QDs/ZnO/Al | 534 | 5.5 | 9140 | This work |
